# Pharmacokinetics and Immunogenicity of Frunevetmab in Osteoarthritic Cats Following Intravenous and Subcutaneous Administration

**DOI:** 10.3389/fvets.2021.687448

**Published:** 2021-06-10

**Authors:** Rodney R. Walters, Joseph F. Boucher, Flavia De Toni

**Affiliations:** Veterinary Medicine Research and Development, Zoetis, Kalamazoo, MI, United States

**Keywords:** feline osteoarthritis, DJD, monoclonal antibody, pharmacokinetics, immunogenicity, anti-drug antibody, SOLENSIA, NV-02

## Abstract

Osteoarthritis and other degenerative joint diseases are common causes of chronic pain in cats. Frunevetmab is a felinized monoclonal antibody that binds to nerve growth factor (NGF) and provides relief from pain by blocking the receptor-mediated signaling cascade induced by NGF. Results from three studies were combined to provide an overview of frunevetmab pharmacokinetics (PK) and immunogenicity. The objective of the first study was to establish the pharmacokinetic parameters resulting from intravenous (IV) and subcutaneous (SC) administration of frunevetmab to the feline patient population at 3 mg/kg. Ten adult cats with naturally-occurring osteoarthritis were administered frunevetmab in a crossover design at 28 day intervals. Non-compartmental pharmacokinetic analysis of the plasma concentration-time data showed that the half-life was 10.1 ± 1.9 days after IV dosing and the SC bioavailability was 60.3 ± 15.8% with maximum drug levels observed at 3–7 days after dosing. Plasma samples were collected at ~28 days after dosing during two field safety and effectiveness studies of cats with degenerative joint disease. The doses ranged from 1.0 to 2.8 mg/kg; 2 or 3 doses were administered either SC/IV, SC/SC, or SC/SC/SC. The data from these studies along with the data from the laboratory pharmacokinetic study were analyzed using non-linear mixed-effects (NLME) modeling. The model closely predicted the trough concentrations from the two field studies, including the IV treatment in the pilot field study. The trough concentrations were predicted to be close to steady-state after 2 doses. A second objective was to determine the incidence and clinical relevance of frunevetmab immunogenicity. A three-tier anti-drug antibody assay (screen, confirm, titer) was developed and validated. Immunogenicity was assessed in 259 frunevetmab-treated animals enrolled in the two field studies. Only 4 of these animals (1.5%) appeared to develop immunogenicity to frunevetmab. None of the four exhibited adverse events attributed to immunogenicity and no impact on drug levels or efficacy was observed in three of the animals. In the placebo animals, 2.3% (3/131) appeared to develop treatment-emergent immunogenicity. Overall, frunevetmab administration resulted in a very low incidence of treatment-emergent immunogenicity with no safety findings and minimal effect on drug exposure and efficacy.

## Introduction

Osteoarthritis (OA) and other degenerative joint diseases (DJD) are prevalent in cats (1). The important role of the neurotrophin beta nerve growth factor (NGF) in chronic pain states, including DJD pain, has been demonstrated in animals and humans (2). NGF binds to its two receptors, tyrosine kinase receptor type 1 (TrkA) and p75, to activate a signaling cascade that triggers neurite outgrowth and sensitization in neurons (3). Several anti-NGF monoclonal antibodies have been tested in human to antagonize NGF activity and provide relief from pain (4–6). In dogs diagnosed with clinical OA, a 0.2 mg/kg intravenous (IV) injection demonstrated some analgesic effect for 4–6 weeks post-injection (7). In cats, a proof-of-concept study and multi-center, placebo-controlled, randomized, double-blind pilot and pivotal field studies demonstrated efficacy of frunevetmab, a felinized anti-NGF mAb (also called NV-02), for the treatment of DJD-associated pain (8–10). Pharmacokinetic data were reported for laboratory cats following single subcutaneous administration of 2.0, 5.6, 16.8, or 28.0 mg/kg frunevetmab to groups of 2 cats (11). Assay of plasma samples collected up to 42 days after dosing showed that peak drug levels were achieved at ~3 days after dosing and the plasma elimination half-life averaged 9 days.

The amino acid sequences of the heavy and light chains of frunevetmab were based on felinization of a rat anti-mouse NGF mAb called αD11 which bound to both murine and feline NGF (11, 12). The sequence of murine NGF is 82% homologous to feline NGF (11). During felinization the complementarity-determining regions (CDRs) of the αD11 antibody were retained and incorporated into antibody constant regions and variable domain frameworks consistent with those of a cat antibody (11). One purpose of felinization was to prevent frunevetmab from being detected as “foreign” by the immune system of cats and thus to prevent an immune response resulting in production of anti-drug antibodies (ADAs). Felinization also ensured that frunevetmab would undergo FcRn recycling, the species-specific receptor-driven process which increases half-life and bioavailability by recycling antibodies which otherwise would be degraded in lysosomes (13). For example, it has been shown in mice that in the absence of FcRn recycling, the subcutaneous bioavailability of a mAb decreased from 76 to 42% and the half-life decreased from ~5 days to 0.5 days (14).

One objective of the current work was to determine the pharmacokinetics of frunevetmab in a laboratory study of a group of ten cats that had been diagnosed with osteoarthritis. The concentration-time data from these cats were also fit to a non-linear mixed-effects (NLME) pharmacokinetic model simultaneously with the concentration-time data from two large repeated-dose field efficacy studies of cats with DJD (9, 10).

The immunogenic potential of frunevetmab was not studied previously. Thus, another objective was to measure anti-frunevetmab ADAs in plasma samples from the PK study and in samples from the previously-completed pilot and pivotal field efficacy studies (9, 10). Non-neutralizing ADAs may cause rapid clearance of a biotherapeutic from circulation and neutralizing ADAs will prevent the binding of a biotherapeutic to its target; in either case the efficacy may be reduced in magnitude or duration (15, 16). Immunogenicity can also result in safety findings, although such findings are rare for antagonistic monoclonal antibody drugs like frunevetmab which are considered to be of low risk since the mode of action is to bind to a soluble cytokine and thereby block the activity of the cytokine (15). Since the incidence of immunogenicity may be low, it is appropriate to study immunogenicity in as many animals as possible.

## Materials and Methods

### Laboratory Pharmacokinetic Study

All experimental procedures involving animals in the laboratory pharmacokinetic study were reviewed in accordance with ethics requirements and authorized by the official ethical committee of ArthroLab Inc. ArthroLab is fully accredited by the Canadian Council on Animal Care (CCAC). Cats were provided by three animal shelters in Quebec, Canada. A formal agreement with the animal shelters allowed ArthroLab to use the animals for non-invasive research purposes and, after completion of research studies, to provide the cats for adoption or to return them to the animal shelters. The animals were of mixed breed, 4 males and 6 females, neutered, weighed 3.3–9.9 kg, and were estimated to be 5–14 years old. Following physical examination, cats deemed healthy, with the exception of a diagnosis of OA, were selected on the basis of age, physical findings (weight, body condition, physical examination findings), absence of clinical pathological findings (CBC and serum chemistry, urinalysis), behavior (not interfering with performance of required procedures), and acclimation (socialization, acceptable pre-study general health and clinical observations, and adequate food consumption). Primary enclosures were as specified by the Canadian Council on Animal Care (17) and in accordance with USDA guidelines (18, 19). Animals were housed in two separate rooms.

A radiographic screening of forelimb (carpus, elbow, shoulder) and hind limb (tarsus, stifle, hip) was used to confirm a diagnosis of radiographic OA. All X-rays were reviewed by a veterinary surgeon. To be selected, a cat had to present radiographic alterations (i.e., presence of osteophytes and/or subchondral sclerosis or cyst) in at least one appendicular joint to be considered as osteoarthritic. Lesions such as meniscal mineralization or enthesiophytes had to be associated with osteophytes and/or subchondral alteration.

Physical examination was conducted using the Montreal Instrument for Cat Arthritis Testing (MI-CAT(V) Short Form) to confirm those with mobility impairment and/or detectable pain associated with OA (20, 21). Neurological assessments, such as pupillary reflexes, flexor withdrawal reflex, extensor postural thrust, knee jerk reflex, and proprioceptive reflex, were conducted prior to dosing and twice during the study.

Frunevetmab injectable solution (7 mg/mL in 5% sorbitol, 10 mM L-histidine, and 0.01% polysorbate 20, pH 6.0) was manufactured at BioNua, Tullamore, County Offaly, Ireland, and stored at 2-8°C.

Animals were randomly assigned to treatment groups with 2 males and 3 females assigned to group A (IV injection on Day 0 followed by SC injection on Day 28) and 2 males and 3 females assigned to group B (SC injection on Day 0 followed by IV injection on day 28). SC injections were administered between the scapulae. All doses were administered at 0.43 mL/kg for a total dose of 3.0 mg/kg.

Blood samples, ~1 mL, were collected by jugular venipuncture at 12 days before the first dose and after dosing at 1 h (IV dose only), 24 h (SC dose only), and 72, 168, 336, 504, and 672 h (IV and SC doses). The blood samples were collected in potassium EDTA tubes, mixed, and placed on wet ice. Plasma was separated by centrifugation and stored frozen until assayed.

### Field Studies

The multi-center, placebo-controlled, randomized, double-blind pilot and pivotal field studies are described in detail elsewhere (9, 10). In summary, in the pilot field study, three groups of 41–43 client-owned cats with DJD were dosed twice with frunevetmab or placebo (9). The placebo group received an IV dose of placebo on Day 0 and a SC dose ~28 days later. A second group similarly received IV and SC doses of frunevetmab (IV/SC). The third group received two SC doses of frunevetmab (SC/SC). Based on the weight of the animal, each cat received 1 or 2 mL of a 7 mg/mL frunevetmab solution formulation such that the actual dose could range from 1.0 to 2.8 mg/kg. Plasma samples for frunevetmab and immunogenicity analysis were collected pre-study, at the end of the first dosing interval (Day 28 ± 3 days), and at the end of the study (Day 56 ± 3 days). The statistical comparison of groups IV/SC and SC/SC showed no meaningful differences with regard to efficacy (using repeated measures analysis of variance), so they were combined for analyses of most efficacy variables (9). Since the incidence of immunogenicity was very low, these two groups were also combined for the analysis of the immunogenicity data.

In the pivotal field study, client-owned cats with DJD were dosed three times with frunevetmab (*n* = 182) or placebo (*n* = 93) (10). All doses were given via the SC route at ~28-day intervals. The actual dose could range from 1.0 to 2.8 mg/kg depending on the weight of the cat. Plasma samples were collected pre-study, at the end of the first and second dosing intervals (Day 28 ± 3 days and Day 56 ± 3 days) and at the end of the study (Day 84 ± 3 days).

### Analysis of Frunevetmab Concentrations

An enzyme-linked immunosorbent assay (ELISA) for the detection of “free” frunevetmab in feline plasma at concentrations from 0.2 to 10 μg/mL was developed and validated at PPD® Laboratories, Richmond, Virginia. The assay was based on the use of murine NGF (Biosensis, catalog # PE-019) to capture frunevetmab via its NGF-binding site and peroxidase-conjugated goat anti-cat IgG (affinity-purified polyclonal antibodies, Jackson ImmunoResearch catalog # 102-035-003) to detect frunevetmab via its heavy and light chain constant regions. In the procedure, an ELISA plate was coated with a solution of NGF, incubated, washed, blocked with assay buffer, and washed again. Duplicate study plasma samples, standards, and controls (20 μL) were diluted 1:200 with assay buffer and a 100 μL volume was added to the wells of an ELISA plate, incubated, and washed. Detection antibody was added, the plate was incubated, washed, and the colorimetric substrate solution added. After additional incubation, stop solution was added to the plate and the optical density was determined using a plate reader. The duplicates were averaged and sample concentrations were determined by interpolation from the standard curve, which was fitted to a 4-parameter logistic curve with 1/response^2^ weighting. The method was fully validated including characterization of accuracy, precision, upper (ULOQ) and lower (LLOQ) quantitation limits, dilutional linearity, prozone effect, specificity, freeze-thaw stability, and up to 31 months storage stability at −80°C.

Although the LLOQ of the assay was 0.2 μg/mL, pre-dose and placebo samples from the field studies frequently appeared to have concentrations as high as 0.5 μg/mL (for example, see **Table 9**). This had no significant impact on the data reported here.

### Non-compartmental Pharmacokinetic Parameter Estimates

The pharmacokinetic parameters of frunevetmab in the laboratory pharmacokinetic study were calculated using Watson LIMS software (ThermoFisher Scientific, version 7.4.1, USA). The elimination rate constant (λ_z_) was determined using least-square regression analysis of the terminal log-linear portion of the plasma concentration profile. Washout was not complete prior to the second dose; therefore, the concentration-time data following the second dose were corrected by subtracting the extrapolated concentrations following the first dosing interval.

Using corrected data, the area under the plasma concentration-time curve (AUC_0−t_ and AUC_0−inf_) was calculated using the linear trapezoid method. Clearance (CL) and absolute bioavailability (F) were calculated using dose-normalized AUC_0−inf_ following SC and/or IV administration: CL = Dose/AUC_0−inf, IV_; F = 100% × (AUC_0−inf, SC_/AUC_0−inf, IV_) × (Dose_IV_/Dose_SC_). The terminal half-life was calculated as t_1/2_ = ln 2/λ_z_. C_0_, the concentration at time zero after IV bolus administration, was estimated by back-extrapolation of the 1 and 72 h concentrations to time zero.

### NLME Model Building

All plasma concentration time-course data collected from the bioavailability, pilot and pivotal field studies were fitted simultaneously using non-linear mixed-effects (NLME) modeling. NONMEM software (version 7.4.3, ICON Plc, Gaithersburg, MD, USA) was used for parameter estimation, sequentially applying the iterative two-stage method followed by the stochastic approximation expectation maximization (SAEM) method and finally the importance sampling (expectation only) estimation method. The pharmacokinetic model was a two-compartment linear PK model. The model was parameterized using an absorption rate constant (ka), systemic clearance (CL), apparent volume of the central compartment (Vc), distributional clearance (Q), apparent volume of the peripheral compartment (Vp) and bioavailability (F). To estimate the between subject variability (BSV), random effects were included for model parameters ka, CL, Vc, Q, and Vp using a full variance-covariance matrix. Bioavailability was originally included for BSV but could not be estimated with an acceptable precision. For clearance and volume parameters body weight was included as a covariate. The specific parameter equations were:

$$\begin{array}{l} CL_{i}=\theta_{CL}\cdot {\left(\frac{bw}{5.4}\right)}^{\theta_{bw,cl}}\cdot e^{\eta_{i}^{\left(CL\right)}}\\ Vc_{i}=\theta_{Vc}\cdot {\left(\frac{bw}{5.4}\right)}^{\theta_{bw,V}}\cdot e^{\eta_{i}^{\left(Vc\right)}}\\ Q_{i}=\theta_{Q}\cdot {\left(\frac{bw}{5.4}\right)}^{\theta_{bw,cl}}\cdot e^{\eta_{i}^{\left(Q\right)}}\\ Vp_{i}=\theta_{Vp}\cdot {\left(\frac{bw}{5.4}\right)}^{\theta_{bw,V}}\cdot e^{\eta_{i}^{\left(Vp\right)}}\\ ka_{i}=\theta_{ka}\cdot e^{\eta_{i}^{\left(ka\right)}}\\ \eta_{i}\sim N\left(0,\Omega \right) \end{array}$$

where θ_xx_ is the population average parameter value for parameter xx at a body weight of 5.4 kg, bw is the baseline body weight of individual i, 5.4 is the median baseline body weight (kg) and $\eta_{i}^{\left(xx\right)}$ is a random deviation from the population average value used to estimate parameter xx for subject i. The vector of η for subject i is assumed to be distributed as multivariate normal with 0 mean and variance-covariance matrix Ω. Additionally an exponential error was used to model the residual variability and was assumed to be normally distributed with mean 0 and standard deviation σ.

### Model Evaluation

Goodness-of-fit plots, including individual and population predicted values vs. observed values, the distributions of conditional weighted residuals (CWRES), and plots for CWRES vs. predicted values and time, were used to assess the model adequacy. Additionally, visual predictive checks (VPC) were used to assess the structural model fit and estimated variability against observed data for the various studies and treatment regimens within study. A VPC was simulated from the fitted model using the actual doses and body weights of the study animals; the median, 5th and 95th percentiles of the simulated data were plotted against the observed data. The expectation was that the median should fall close to the center of the data at each time point and that 90% of the individual concentration values were contained within the 5th and 95th percentiles.

Monte Carlo simulation was used to compare dose-normalized observed trough levels from the pilot and pivotal studies with the model predicted values. These simulations took into account the actual body weights of the cats in these studies, but did not take into account variability in the dosing interval, which was assumed to be 28 days. Plotting this prediction distribution against observations of the concentrations from samples collected in the field studies gave additional insight into the quality of the predictive ability of the model.

### Analysis of Anti-frunevetmab Antibodies

Ligand-binding assays for the detection of anti-drug antibodies (ADAs) to frunevetmab in feline plasma were developed and validated at PPD® Laboratories, Richmond, Virginia. The three tiers of assay consisted of screening, confirmatory, and titer assays (22). The assays were based on the use of biotin-labeled frunevetmab to capture anti-frunevetmab ADAs and ruthenium-labeled frunevetmab to detect the ADAs by electrochemiluminescence using a Meso Scale Discovery® (MSD) instrument. The assays included an acid dissociation step to dissociate frunevetmab-ADA complexes and thus increase the drug tolerance of the assay (23). A humanized anti-NGF monoclonal antibody (with a CDR sequence different from frunevetmab; prepared in-house) was added to bind NGF and prevent target interference (23). The positive control (PC) for the assays was affinity-purified rabbit polyclonal anti-frunevetmab antibodies (prepared in-house) and diluted into blank feline plasma. The negative control (NC) was blank feline plasma.

In the screening assay, a 20 μL aliquot of study sample, NC, or PC plasma was mixed with an equal volume of assay buffer and 360 μL of 300 mM acetic acid (hence the minimum required dilution, MRD, of the assay was 20). After incubation to dissociate frunevetmab-ADA complexes, 50 μL were transferred to the wells of a plate which contained 90 μL of reaction solution (0.25 μg/mL biotin-frunevetmab, 0.25 μg/mL ruthenium-frunevetmab, and 30 μg/mL humanized anti-NGF antibody in assay buffer) and 15 μL of neutralizing solution (1 M Tris-HCl, pH 9.5). After incubation to allow the ADAs to bind to the capture and detection reagents, a 50 μL aliquot of each sample was transferred to a blocked streptavidin-MSD plate in duplicate. After incubation and washing, 2X Read Buffer T was added to each well and the plate was read on an MSD detector. The method was fully validated including evaluation of matrix interference, drug tolerance, drug target interference, intra- and inter-assay precision, prozone effect, freeze/thaw stability, effect of hemolysis, and stability at room temperature and −80°C storage. The normalized screening cut point, 1.06, calculated as the ratio of the signal of the sample divided by the average signal of the negative control plasma, was determined statistically from the analysis of 50 individual drug-naïve feline plasma samples over 6 independent assay runs. A 5% false positive incidence was targeted (22), after removal of 4% of the samples that were biological outliers. The screening assay sensitivity was 35.5 ng/mL ADAs in the absence of frunevetmab. PC plasma samples with ADA concentrations of 51, 100, and 500 ng/mL were assayed within-day and day-to-day with ≤10.9% coefficients of variation. The drug tolerance was 26 μg/mL frunevetmab; at this concentration 98 ng/mL ADAs could still be detected.

The confirmatory assay was the same, except a second 50 μL aliquot of acid-dissociated sample was transferred to wells which contained 90 μL of inhibition reaction solution (0.25 μg/mL biotin-frunevetmab, 0.25 μg/mL ruthenium-frunevetmab, 30 μg/mL humanized anti-NGF antibody, and 30 μg/mL frunevetmab in assay buffer) and 15 μL of neutralizing solution (1 M Tris-HCl, pH 9.5). The cut point, 34.1% inhibition, was determined statistically from the analysis of 50 individual drug-naïve feline plasma samples over 6 independent assay runs. A 1% false positive incidence was targeted (22), after removal of 1.3% of the samples that were biological outliers. PC plasma samples with ADA concentrations of 51, 100, and 500 ng/mL were assayed within-day and day-to-day with ≤9.75% coefficients of variation.

The titer assay utilized the same procedure as the screening assay but with each sample diluted in 2-fold steps with NC plasma until a negative response was obtained. The titer was reported as the reciprocal of the last dilution with a response above the cut point. The titer cut point (for titer assays of confirmed-positive samples) was 1.17, based on the same statistical analysis as for the screening cut point, but targeting 0.1% false positives (22) after removal of 4% of the samples that were biological outliers.

### Immunogenicity Classification

The ADA data from each animal were used to classify the immunogenicity status of the animal into one of four categories: no immunogenicity (none of time points were confirmed-positive); pre-existing reactivity that was not boosted (the titer did not increase more than 2-fold at any post-dose time point relative to the pre-dose time point); treatment-boosted immunogenicity (the titer increased more than 2-fold post-dose relative to the pre-dose time point); or treatment-induced immunogenicity (the pre-dose sample was negative in the screening or confirmatory assay, but at least one post-dose time point was confirmed-positive with a titer ≥MRD) (24). These classifications were based on industry standards and the latter two classifications (treatment-boosted and treatment-induced) together constituted the animals with treatment-emergent immunogenicity (25).

### Characterization of Immunogenicity

To determine the clinical relevance of immunogenicity findings, the ADA data were interpreted using the frunevetmab concentration data, efficacy data, and adverse events data. The primary efficacy parameter was the client-specific outcome measures (CSOM) treatment success, which was evaluated based on a cat's ability to perform individually-tailored activities (9, 10).

## Results

### OA Evaluation of Animals in the Laboratory Pharmacokinetic Study

Radiographic screening demonstrated the presence of OA in at least one appendicular joint in each cat. Physical examinations conducted using the MI-CAT(V) Short Form demonstrated clinical signs of mobility impairment and/or pain associated with OA (Table 1).

**Table 1:** MI-CAT(V) evaluation of the study animals.

**Cat**	**MI-CAT(V) Evaluation**
19-001 (male)	45.1%
19-002 (male)	66.7%
19-003 (male)	41.9%
19-004 (male)	42.7%
19-005 (female)	40.7%
19-006 (female)	52.4%
19-007 (female)	25.6%
19-008 (female)	57.7%
19-009 (female)	24.8%
19-010 (female)	53.7%

### Adverse Events

No serious or non-serious adverse events occurred during the laboratory pharmacokinetic study.

### Frunevetmab Concentration-Time Profiles

**Figure 1:**
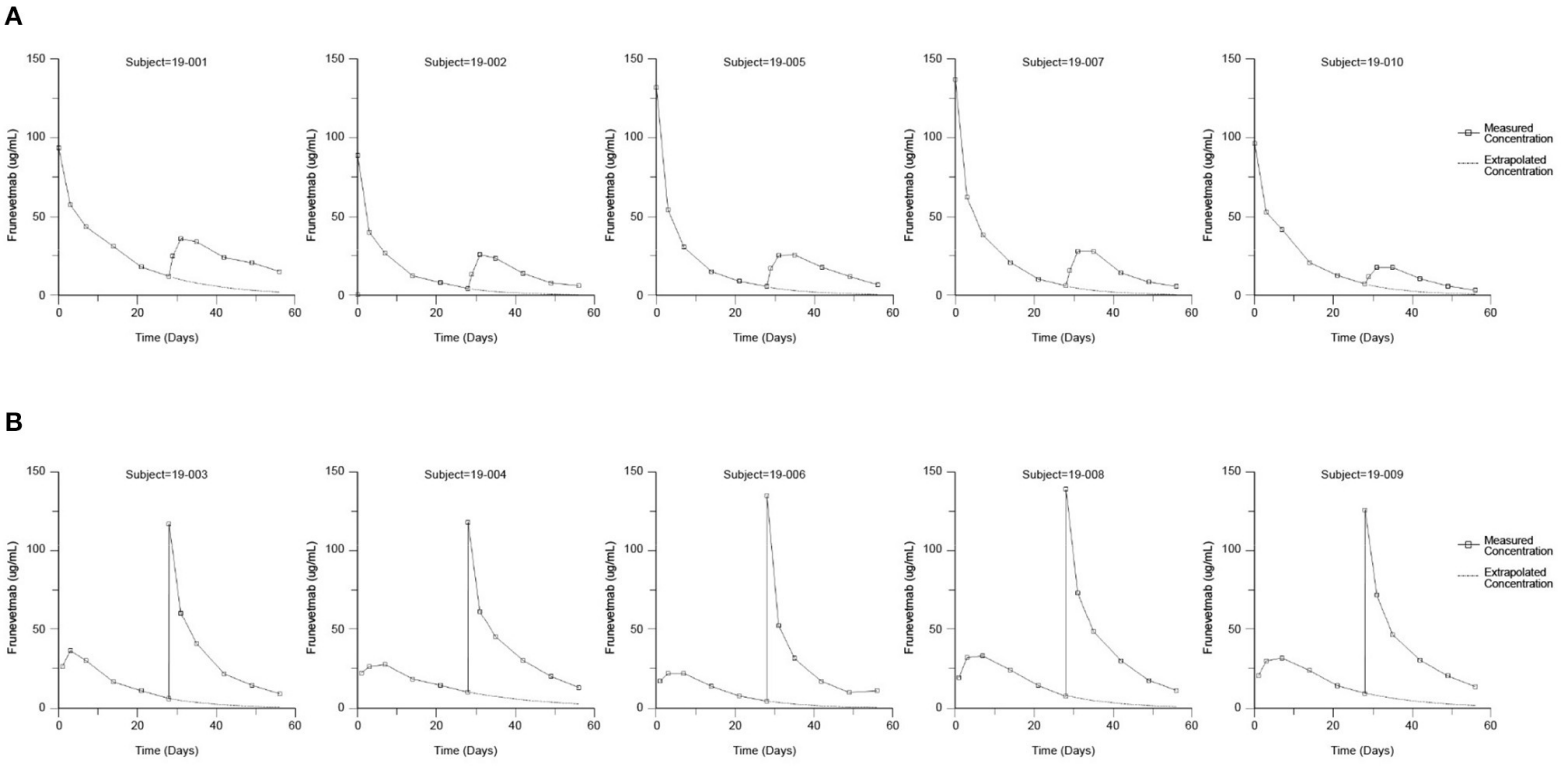
Measured frunevetmab concentrations after 3.0 mg/kg administration on Days 0 and 28. Administration order: IV followed by SC **(A)** or SC followed by IV **(B)**. The extrapolated concentrations from the first dose are shown as dashed lines.

**Figure 2:**
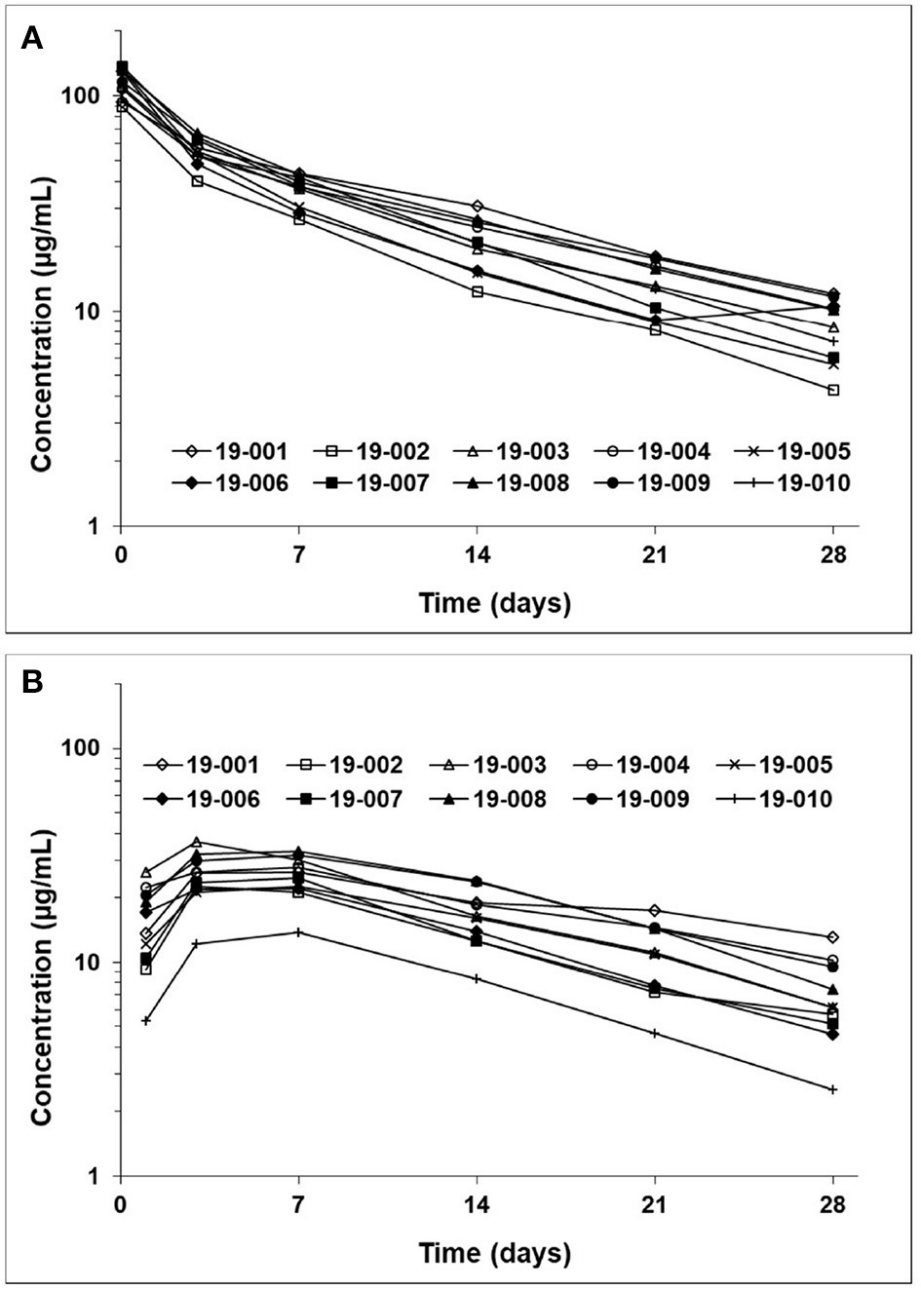
Frunevetmab corrected concentration-time profiles after IV **(A)** and SC **(B)** administration at 3.0 mg/kg.

**Figure 3:**
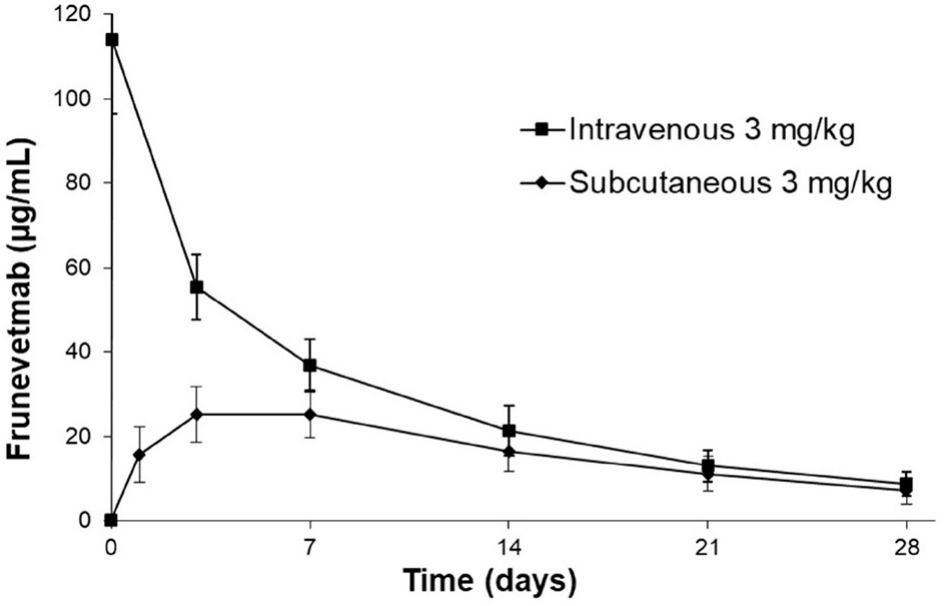
Average frunevetmab concentrations (± SD) in ten cats diagnosed with OA.

The frunevetmab concentration-time profiles are shown in Figure 1. The dosing interval, 28 days, was not sufficient to completely wash out the drug prior to the second dose. Therefore, to avoid over-estimating C_max_ and AUC after the second dose, the concentration-time data for the second dosing period were corrected by subtracting the extrapolated concentrations from the first dosing period (see also Figure 1). The individual concentration-time profiles, after correction, are shown in Figure 2 and the average concentrations ± SD are in Figure 3.

### Frunevetmab Non-compartmental Pharmacokinetic Parameter Estimates

After IV administration, there was an initial distribution phase which was not well-characterized (Figure 3). The terminal elimination phase was evaluated from days 7 to 28. The non-compartmental pharmacokinetic results are shown in Table 2.

**Table 2:** Frunevetmab non-compartmental pharmacokinetic parameters (mean ± SD; *n* = 10) for cats with OA after IV and SC administration at 3.0 mg/kg.

**Parameter**	**IV Route**	**SC Route**
C_0_ (μg/mL)	116 ± 18	–
C_max_ (μg/mL)	–	26.1 ± 6.5
t_max_ (d)	–	3–7 (range)
λ_z_ (d^−1^)	0.0708 ± 0.0128	0.0638 ± 0.0154
t_1/2_ (d)	10.1 ± 1.9	11.7 ± 4.2
CL (mL/d/kg)	3.19 ± 0.66	–
AUC_0−inf_ (d^*^μg/mL)	970 ± 169	590 ± 209
F (%)	–	60.3 ± 15.8
C_28days_ (μg/mL)	8.65 ± 2.75	7.05 ± 3.10

### Frunevetmab Trough Concentrations in Field Studies

Table 3 summarizes the frunevetmab concentration data from the samples collected prior to each dose and at the end of the pilot and pivotal field studies (9, 10). Dosing was scheduled every 28 ± 3 days; thus the samples were collected from 25 to 31 days after each dose. In the pivotal field study, the animals received three SC doses of frunevetmab every 28 ± 3 days (10). The individual animals received doses in the range of 1.0–2.8 mg/kg. The data in Table 3 were not dose-normalized. The results summarized in Table 3 excluded animals classified as having treatment-emergent immunogenicity as well as animals that were non-evaluable because samples were not collected during the scheduled visits.

**Table 3:** Frunevetmab concentrations (mean ± SD) at the end of each dosing interval in field studies conducted at 1.0–2.8 mg/kg.

**Study**	**Concentration (μg/mL) on study day (Targeted dosing interval: 28 days)**		
	**28 ± 3 days**	**56 ± 3 days**	**84 ± 3 days**
Pilot field study; Group IV/SC (*n =* 39^‡^)	5.77 ± 2.86	5.35 ± 3.47	–
Pilot field study; Group SC/SC (*n =* 39^‡^)	4.02 ± 2.39	5.02 ± 3.93	–
Pivotal field study; Frunevetmab group (*n =* 177^‡^)	4.12 ± 2.54	4.96 ± 3.63	5.12 ± 3.72

‡ *Excluding non-evaluable animals and animals with immunogenicity*.

### Pharmacokinetic Model

**Figure 4:**
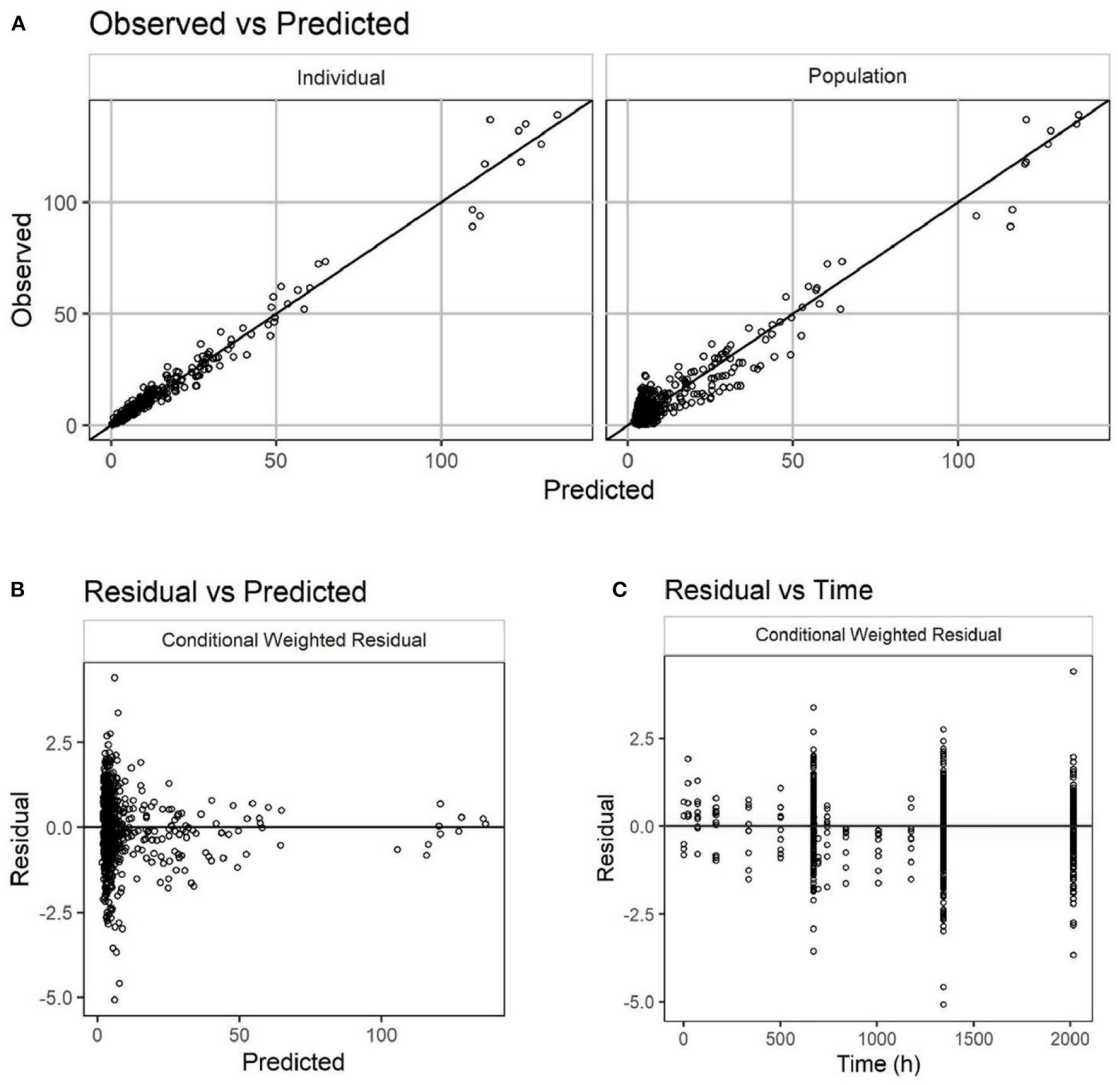
Goodness-of-fit plots for the PK model of frunevetmab concentrations (units of both axes: μg/mL). Observed vs. predicted concentrations **(A)**; Residual vs. predicted concentrations **(B)**; Residual concentrations vs. time **(C)**.

**Figure 5:**
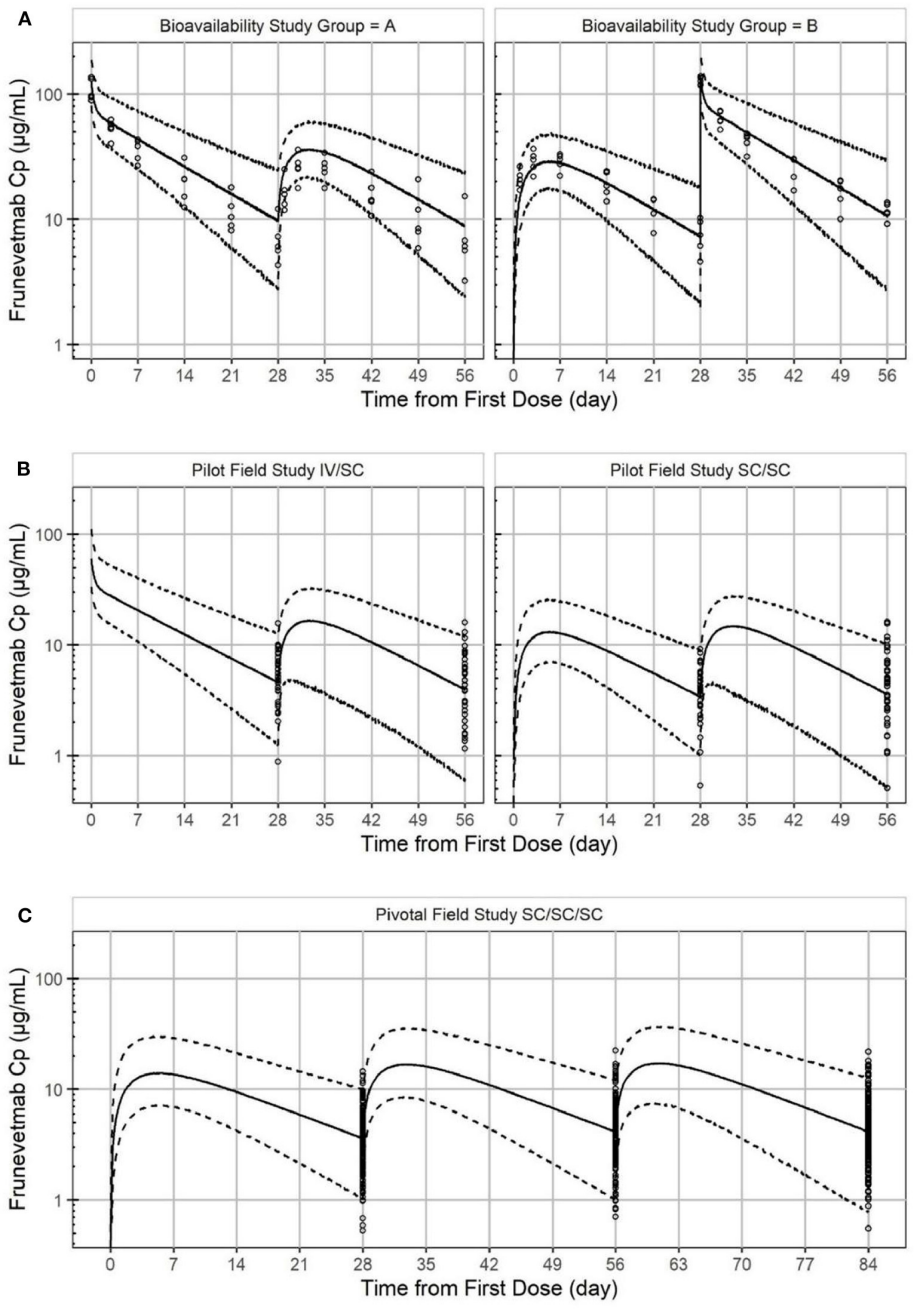
Visual predictive check of frunevetmab concentration-time profiles showing the observed data plotted against the median simulated concentration (solid line) from the PK model with a 95% prediction interval (dashed line) for the laboratory pharmacokinetic study **(A)**, the pilot field study **(B)**, and the pivotal field study **(C)**.

A total of 777 plasma concentration samples from 265 cats were simultaneously modeled using NLME. Missing samples, samples below the lower limit of quantitation, samples from non-evaluable animals, and samples from animals with immunogenicity were not included. The laboratory pharmacokinetic study had full IV and SC concentration-time profiles for 10 cats while the pilot and pivotal field studies measured only trough concentrations. A two-compartment linear PK model with first order absorption was found to well describe the PK of frunevetmab in cats. Goodness of fit plots are shown in Figure 4 and show no major concerns. The residual vs. predicted plot does show a funnel pattern; this is, however, a reflection of the abundance of data points taken at the trough and not a mis-specified model. Visual predictive checks (VPC), in which at least 100 simulations of each study animal were generated and pooled, showed good representation of both the central tendency and variability associated with frunevetmab plasma concentrations (Cp) of each study subgroup (Figure 5).

### NLME Parameter Estimates

Final parameter estimates can be found in Table 4. The imprecision of the PK parameter estimates [relative standard error (RSE)] from the final NLME model was highest for the absorption rate constant (69.6%) and the distributional clearance (67.4%). This may be expected in that only the bioavailability study had information available to estimate these parameters. The between subject variability (BSV) ranged from 6.37 to 33.4% with the highest BSV for CL. Overall, the population clearance estimate was 0.648 mL/h and the estimated apparent volumes of distribution were 0.127 L and 0.0852 L for the volumes of the central and the peripheral compartments, respectively. The population absolute bioavailability of SC frunevetmab (F_SC_) was estimated at 59.2% which was consistent with the noncompartmental result from the bioavailability study.

**Table 4:** Parameter estimates for two-compartment mixed effect model of frunevetmab plasma concentrations.

**Parameter**	**Estimate (SE)**	**RSE (%)**	**BSV (% CV)**
Ka (1/h)	0.0139 (0.00968)	69.6	27.9
CL (mL/h)	0.648 (0.0954)	14.7	33.4
Vc (L)	0.127 (0.0422)	33.2	11.3
Q (mL/h)	5.27 (3.55)	67.4	28.3
Vp (L)	0.0852 (0.00265)	3.11	6.37
F	0.592 (0.0283)	4.78	NA
Θ_bw,cl_	1.18 (0.0121)	1.03	NA
Θ_bw,V_	1.29 (0.00272)	0.211	NA
Exponential Residual Error (% CV)	25.4		

### NLME Model Predictions of Dose-Normalized Frunevetmab Trough Concentrations in Field Studies

The PK model was used to simulate animals with the same dosing regimen used in the pilot and pivotal field studies covering the observed body weight range, however, all doses were held fixed at 1 mg/kg. Comparing the simulated trough concentrations to the dose-normalized observed values (Table 5) indicated very good agreement. Means of the simulated data tended to be nominally less than the observed with comparable standard deviations. Some accumulation of drug from the first to the second doses was predicted; very little additional accumulation was predicted for the third dose. The overall accumulation of the trough concentrations was predicted to be 24% (Table 5).

**Table 5:** Predicted and measured dose-normalized frunevetmab concentrations at the end of each dosing interval in field studies.

**Study**		**Dose-normalized concentration (μg/mL at 1 mg/kg dose)**		
		**Day 28**	**Day 56**	**Day 84**
Pilot field study; Group IV/SC (*n =* 39^‡^)	Predicted	3.75 ± 2.34	3.26 ± 2.32	–
	Observed	3.95 ± 1.80	3.66 ± 2.07	–
Pilot field study; Group SC/SC (*n =* 39^‡^)	Predicted	2.77 ± 1.64	3.02 ± 2.16	–
	Observed	2.89 ± 1.58	3.67 ± 2.72	–
Pivotal field study; Frunevetmab group (*n =* 177^‡^)	Predicted	2.71 ± 1.43	3.19 ± 1.93	3.21 ± 2.11
	Observed	2.72 ± 1.52	3.28 ± 2.23	3.38 ± 2.25

‡ *Excluding non-evaluable animals and the animals listed in Table 9*.

### NLME Model Predictions of Dose-Normalized Frunevetmab Steady-State Pharmacokinetics

Since the field studies did not assess PK parameters such as C_max_, the PK model was also used to simulate the steady-state subcutaneous pharmacokinetic parameters at a dose of 1 mg/kg (Table 6). The simulation included the same dosing regimen used in the pilot and pivotal field studies covering the observed body weight range, however, all doses were held fixed at 1 mg/kg.

**Table 6:** Predicted steady-state subcutaneous pharmacokinetic parameters for frunevetmab at 1 mg/kg.

**Parameter**	**Prediction at steady-state (1 mg/kg dose; Mean and 90% prediction Interval)**
C_max_ (μg/mL)	12.2 (8.34–17.7)
t_max_ (d)	5.0 (3.5–6.6)
t_1/2_ (d)	10.3 (6.17–15.6)
AUC_0−28*d*_ (d*μg/mL)	218 (121–356)

### Immunogenicity

The samples from all three studies were tested using the ADA assays (Table 7) and the immunogenicity classification of each animal was determined (Table 8). In one of the studies an in-study normalized screening cut point was utilized (1.09 instead of 1.06; Table 7) due to the high incidence of screen positives in the pre-dose samples. None of the animals from the laboratory pharmacokinetic study exhibited treatment-emergent immunogenicity. In the two field studies combined (totalling 259 drug-treated and 131 placebo-treated animals), three drug-treated animals were classified as having developed treatment-emergent immunogenicity. In addition, one drug-treated animal, classified as having pre-existing reactivity that was not boosted, had low drug levels and poor CSOM efficacy. The data used to characterize the ADAs from these four animals (4/259, 1.5%) are listed in Table 9. Three of the placebo-treated animals in the field studies appeared to develop treatment-emergent immunogenicity (3/131, 2.3%; Table 9).

**Table 7:** Results of ADA analyses by sample (% and number of samples).

**Study**	**Group**	**Pre-dose screen positive**	**Pre-dose confirmed positive**	**Post-dose screen positive**	**Post-dose confirmed positive**
Laboratory pharmacokinetic study	Frunevetmab (*n =* 10 animals)	30.0% (3/10)	10.0% (1/10)	21.7% (26/120)	0.0% (0/120)
Pilot field study	Placebo (*n =* 39 animals)	18.4% (7/38)^‡^	13.2% (5/38)	25.4% (18/71)^‡^	12.7% (9/71)
	Frunevetmab^†^ (*n =* 80 animals)	20.3% (16/79)^‡^	12.7% (10/79)	20.0% (31/155)^‡^	6.5% (10/155)
Pivotal field study	Placebo (*n =* 92 animals)	20.7% (19/92)	6.5% (6/92)	30.6% (71/232)	9.1% (21/232)
	Frunevetmab (*n =* 179 animals)	14.0% (25/179)	5.6% (10/179)	24.3% (115/474)	3.8% (18/474)

† *IV/SC and SC/SC combined*.

‡ *In-study screening cut point = 1.09*.

**Table 8:** Immunogenicity classification by animal.

		**Immunogenicity classification**			
**Study**	**Group**	**No immunogenicity**	**Pre-existing reactivity that was not boosted**	**Treatment-boosted ADAs**	**Treatment-induced ADAs**
Laboratory pharmacokinetic study	Frunevetmab (*n =* 10)	9	1	0	0
Pilot field study	Placebo (*n =* 39)	34	5	0	0
	Frunevetmab^†^ (*n =* 80)	69	9	1	1
Pivotal field study	Placebo (*n =* 92)	84	5	1	2
	Frunevetmab (*n =* 179)	168	10	0	1

† *IV/SC and SC/SC combined*.

**Table 9:** Characterization of treatment-emergent immunogenicity in field studies.

**Study**	**Group**	**Animal**	**Visit**	**Frunevetmab (μg/mL)**	**Immunogenicity Assays**			**CSOM success**
					**Screen result**	**Confirmatory result**	**Titer**	
Pilot	Frunevetmab	1	0	0.222	Detected	Positive	20	–
			28	8.03	Detected	Positive	20	Yes
			56	6.86	Detected	Positive	320	Yes
Pilot	Frunevetmab	2	0	0.338	Detected	Negative	Not Assayed	–
			28	1.34	Detected	Positive	80	Yes
			56	2.30	Detected	Positive	80	Yes
Pivotal	Frunevetmab	3	0	0.407	Negative	Not Assayed	Not Assayed	–
			28	4.27	Detected	Negative	Not Assayed	Yes
			56	7.18	Detected	Positive	20	Yes
			84	6.21	Detected	Positive	20	Yes
Pivotal	Frunevetmab	4^‡^	0	0.210	Detected	Positive	320	–
			28	BLQ	Detected	Positive	160	No
			56	BLQ	Detected	Positive	320	No
			84	0.320	Detected	Positive	40	No
Pivotal	Placebo	5	0	BLQ	Detected	Positive	80	–
			28	BLQ	Detected	Positive	320	Yes
			56	BLQ	Detected	Positive	80	Yes
			84	BLQ	Detected	Positive	320	Yes
Pivotal	Placebo	6	0	0.228	Detected	Negative	Not Assayed	–
			28	0.227	Detected	Negative	Not Assayed	No
			56	0.287	Detected	Positive	80	No
			84	0.356	Detected	Negative	NA	Yes
Pivotal	Placebo	7	0	BLQ	Detected	Positive	<20	–
			28	BLQ	Detected	Positive	40	No
			56	BLQ	Detected	Positive	40	No
			84	BLQ	Detected	Positive	40	Yes

*BLQ, Below the lower limit of quantitation, 0.20 μg/mL*.

‡ *Classified as having pre-existing reactivity that was not boosted following drug administration, but included in this table due to observed low drug levels*.

## Discussion

### Pharmacokinetics

An assay for frunevetmab in feline plasma was developed and validated based on the use of NGF to capture drug and a labeled anti-canine IgG antibody for detection. It is important to note that since the assay was based on the capture of frunevetmab by the drug target, NGF, the assay was a “free” drug assay, meaning that only frunevetmab that was still capable of binding NGF would be detected (26). Thus, frunevetmab bound to NGF or to neutralizing ADAs would not be detected in the assay.

In a previous study, frunevetmab pharmacokinetic data were reported following single subcutaneous administration of 2.0, 5.6, 16.8, or 28.0 mg/kg frunevetmab to groups of 2 cats (11). Peak drug levels were achieved at ~3 days after dosing (range 1.9–4.3 days) and the plasma elimination half-life averaged 9 days (range 7–15 days). The results of the current study (Table 2) confirmed similar pharmacokinetic behavior in a group of 10 cats diagnosed with OA. From non-compartmental analysis after IV administration at 3 mg/kg, the plasma elimination half-life averaged 10.1 ± 1.9 days (range 7.8–13.7 days) and after SC administration peak drug levels were observed at 3–7 days after dosing, the plasma elimination half-life averaged 11.7 ± 4.2 days (range 8.6–22 days), and the bioavailability was 60.3 ± 15.8% (range 27.6–83.4%). These results support the planned once-monthly dosing interval and show that the bioavailability is adequate to permit subcutaneous administration.

Since the 28-day dosing interval was not sufficient to completely wash out the drug prior to the second dose, the non-compartmental parameters were calculated after correcting data from the second dosing period by subtracting the extrapolated concentrations from the first dosing period (see also Figure 1). This correction was justified based on data from other studies of frunevetmab, which showed that frunevetmab exhibited nearly linear pharmacokinetics over a wide dose range. Furthermore, the correction was relatively small, accounting for subtraction of an average of 13% of the AUC of the second dosing profile.

Non-linear mixed-effects (NLME) modeling of all the data from the three studies was able to fit both plasma concentration-time curves and also quantified the variability. The model closely predicted the trough concentrations from the two field studies, including the IV treatment in the pilot field study (Table 5). An additional source of variability that was not accounted for in the model was the deviation from the targeted blood sampling time of 28 days after each dose.

Some of the parameters had considerable η-shrinkage indicating that parameter estimates for individual animals tended to regress toward the population average estimate. This is expected with mixed models especially when some individuals have only a small amount of data, as in the field study data. The field study cats had only trough values measured and thus contributed little to the estimation of absorption or distribution. This was reflected in the η-shrinkage for parameters ka and Q, both of which were close to 50%.

### Immunogenicity

Since detailed guidance for evaluation of the immunogenicity of animal health biotherapeutics is not available, the risk-based process used in human health was applied to the veterinary studies in this report with appropriate analytical methods and immunogenicity characterization (22, 27–29). The three-tier assay for frunevetmab ADAs was developed and validated with statistically-determined cut points. The normalized screening cut point for frunevetmab ADAs, 1.06, was determined from repeated analysis of 50 individual drug-naïve feline plasma samples over 6 independent assay runs. To maximize detection of ADAs, the screening cut point was established such that 5% false positives were expected (22). The confirmatory cut point was similarly determined to be 34.1% inhibition with a 1% false positive incidence targeted. The “true” false positive incidences for both tiers of the assay were higher since biological outliers were removed prior to the cut point calculation (see Table 7—the percentages of pre-dose screen and confirmed positives were substantially >5 and 1%, respectively). The third tier of the ADA assay was a titer determination using 2-fold or 3-fold dilution steps (in negative control plasma). The titer cut point was 1.17, based on the same statistical analysis as for the screening cut point, but targeting 0.1% false positives (22).

Using a surrogate positive control sample prepared by diluting affinity-purified anti-frunevetmab antibodies from immunized rabbits into cat plasma (23, 25), the screening assay sensitivity was determined to be 35.5 ng/mL in the absence of frunevetmab. The assay incorporated an acid dissociation step to improve drug tolerance; 100 ng/mL of ADAs could be detected in the presence of up to 26 μg/mL frunevetmab. Thus, the drug tolerance was adequate to detect ADAs in all of the samples from the field studies and most of the samples from the laboratory pharmacokinetic study. A humanized anti-NGF mAb was added to the assay mixture to bind NGF (30). Since NGF occurs naturally as a homodimer, it can cross-link biotin-labeled frunevetmab and ruthenium-labeled frunevetmab in the same manner as ADAs and thereby cause false-positive results. It was important to choose a mAb with constant and variable regions that were different from frunevetmab so that the added mAb would not bind to anti-frunevetmab ADAs but would bind NGF and prevent NGF from interfering in the assay.

In the pilot and pivotal field studies, “trough” samples were collected for ADA analysis at the end of each 28-day dosing interval as well as prior to the first dose and at the end of the study, 28 days after the last dose. The end of each dosing interval was the most appropriate time to collect samples for ADA analysis in a repeated-dose study since the drug concentration was the lowest and the possibility of interference in the detection of ADAs by drug in the samples was minimized (22). Also, the end of the dosing interval was optimal for detection of any potential persistent immune response which may have developed and matured with time after dosing (24). In addition, the trough drug concentrations in these samples aided in characterizing ADAs based on a determination of whether a sufficient concentration of “free” drug remained in circulation throughout the dosing interval.

In the three studies of cats with DJD, the screen positive incidence in the pre-dose samples ranged from 14.0 to 30.0% (Table 7). This was higher than the 2–11% screening positive incidence that is generally considered acceptable (31). The confirmatory assay reduced the incidence in the pre-dose samples to 5.6–13.2% (Table 7), although this was still much higher than the targeted 1% for the confirmatory assay. However, most of the animals with pre-dose confirmed positive samples did not exhibit an increase in titer following frunevetmab administration; therefore, most of these animals were classified as having “pre-existing reactivity that was not boosted” following drug administration (Table 8).

Pre-existing responses in samples tested for ADAs may be due to antibodies that are not specific to the therapeutic or due to some type of assay interference (32–35). Common types of pre-existing antibodies include rheumatoid factor, anti-allotype antibodies, and anti-glycan antibodies. In most cases these antibodies are not boosted after drug administration and the antibodies do not clear or neutralize the drug. Common types of non-antibody pre-existing reactivity include acquired polyreactivity due to the ADA assay acid treatment, enhanced binding of assay reagents to matrix components, and surface effects from assay beads or plates resulting in protein unfolding during the assay.

Due to biological and analytical variability, the observed pre-existing reactivity may fluctuate over time. An upward fluctuation resulting in a titer increasing more than one dilution step can result in animals being classified as having treatment-boosted or treatment-induced immunogenicity. This can occur in placebo animals as well as in drug-treated animals, for example, 3 of the placebo animals in the pivotal field study were classified as having treatment-emergent immunogenicity (Table 9). When there are similar findings in drug-treated animals, it cannot be definitively determined whether the findings are false positives or true immune responses; thus characterization of the results becomes critical for assessment of the clinical relevance of the findings.

Table 8 shows that 20 drug-treated animals were classified as having pre-existing reactivity that was not boosted. The frunevetmab concentration data and efficacy data (when available) were reviewed to characterize the clinical relevance of the pre-existing reactivity. One animal (animal 4 in Table 9) was identified with very low trough drug concentrations and high ADA titers (but which did not increase over the course of the study), and no CSOM treatment success at any time point. This animal did show a considerable decrease in DJD pain based on veterinary orthopedic examinations from a score of 43 on day 0 to scores of 32, 23, and 28 on the day 28, 56, and 84 visits, respectively (10). Although this animal may simply have exhibited poorer PK characteristics (more rapid clearance and/or poorer subcutaneous bioavailability) than other animals, it was included in the overall total of animals with treatment-emergent immunogenicity since the cause of the poor drug exposure could not be definitively determined. This animal did not exhibit any adverse events attributed to immunogenicity.

A total of 3 drug-treated animals in the field studies were classified as having treatment-boosted or treatment-induced immunogenicity (Table 8). As shown in Table 9, the frunevetmab concentrations of drug-treated animals 1–3 were comparable to the main group of animals without immunogenicity (compare to Table 3), suggesting that the ADAs in these 3 animals were not neutralizing or clearing. These animals also exhibited treatment success at all post-dose time points (Table 9). None of the three exhibited adverse events attributed to immunogenicity.

Overall, the incidences of treatment-emergent immunogenicity in the field studies were reported to be 1.5% (4/259) of the frunevetmab-treated animals (including the animal discussed above with pre-existing reactivity that was not boosted) and 2.3% (3/131) of the placebo animals. The similar incidences in the drug-treated and placebo animals suggests that most of these findings may have been false positives. This was supported in the frunevetmab-treated animals by the minimal effects of the ADAs on drug exposure and efficacy and the absence of adverse effects.

Thus, although frunevetmab, like any other monoclonal antibody product, may induce anti-drug antibodies which may reduce the efficacy of the product, the findings from 259 frunevetmab-treated animals monitored in the pilot and pivotal field studies of duration up to 84 days and from 10 animals in the laboratory pharmacokinetic study showed that frunevetmab has a low potential for inducing clinically relevant immunogenicity.

## Data Availability Statement

The raw data supporting the conclusions of this article will be made available by the authors, without undue reservation.

## Ethics Statement

The animal study was reviewed and approved by the ethical committee of ArthroLab Inc. Arthrolab Inc. is fully accredited by the Canadian Council on Animal Care (CCAC).

## Author Contributions

RW participated in the design and execution of the studies and wrote the main portion of the manuscript. RW, FD, and JB participated in the data analysis and interpretation and drafting and revising of the manuscript. All authors endorse the content of the work.

## Conflict of Interest

RW, JB, and FD were employed by the company Zoetis. The authors declare that this study received funding from Zoetis.
